# Basic Life Support Knowledge and Simulated Chest Compression Performance Among Primary Health Care Staff: A Multicentre Cross-Sectional Study

**DOI:** 10.3390/jcm15124460

**Published:** 2026-06-09

**Authors:** Rafał Wójcik, Tomasz Kłosiewicz, Mateusz Puślecki

**Affiliations:** Department of Medical Rescue, Poznan University of Medical Sciences, 7 Rokietnicka Street, 60-608 Poznań, Poland; tklosiewicz@ump.edu.pl (T.K.); mateuszpuslecki@o2.pl (M.P.)

**Keywords:** basic life support, cardiopulmonary resuscitation, primary health care, patient safety, out-of-hospital cardiac arrest, staff development, psychomotor performance

## Abstract

**Background**: Out-of-hospital cardiac arrest (OHCA) remains a major public health problem. Many patients contact primary health care (PHC) services shortly before cardiac arrest, yet data on PHC staff preparedness to provide guideline-concordant basic life support (BLS) remain limited. This study assessed BLS knowledge and chest compression quality among medical and non-medical PHC staff. **Methods**: This multicentre cross-sectional simulation-based study was conducted in Poznań and Poznań County, Poland. PHC staff with direct patient contact were included (n = 162). Assessment comprised an author-developed 15-item knowledge test based on European Resuscitation Council guidelines and a two-minute continuous chest compression trial on a Resusci Anne QCPR manikin. Correlations were analysed using Spearman’s rank correlation coefficient, group differences using the Kruskal–Wallis test with Dunn–Bonferroni post hoc comparisons, and predictors using multivariable linear regression. **Results**: The median BLS knowledge score was 9/15 points (mean 8.74). Mean chest compression depth was 41.3 mm, below the recommended range, with only 23.5% of compressions meeting depth criteria. Correct compression rate was maintained in 30.2% of compressions, and full chest recoil was observed in 55.0% of attempts. Age was negatively correlated with compression rate. In participant-level regression, higher BLS knowledge was associated with better QCPR performance; however, this association was attenuated and no longer statistically significant in mixed-effects models accounting for clustering by practice. **Conclusions**: PHC staff demonstrated gaps in BLS knowledge and inadequate simulated chest compression performance, particularly regarding compression depth and rate. These findings support recurrent, simulation-based BLS training for all PHC personnel.

## 1. Introduction

Out-of-hospital cardiac arrest (OHCA) is defined as the cessation of effective mechanical cardiac activity, manifested by the absence of signs of circulation, occurring outside the hospital setting [1]. The incidence of OHCA confirmed by emergency medical services in Europe is estimated at 47.8–57.9 per 100,000 inhabitants [2]. In Poland, the incidence of OHCA in which resuscitation attempts were initiated was reported as 69.7 per 100,000 inhabitants in 2018 [3].

The risk of sudden cardiac arrest increases with age and the presence of chronic diseases, particularly cardiovascular conditions [4]. Individuals aged ≥65 years account for approximately 36% of patients attending primary health care practices in Poland [5]. Across Europe, 84% of people aged ≥65 years consulted a PHC physician within the previous year [6]. Importantly, 25% of patients had contact with PHC services in the year preceding OHCA, and this proportion increased to 42% during the week before cardiac arrest [7].

No studies have been published on the frequency of cardiac arrest occurring in primary care practices in Poland. For the European population, the incidence of cardiac arrest in general practice has been estimated at 15.3–79.0 per 100,000 inhabitants per year [8]. The lack of national data hampers assessment of the scale of the problem within the Polish health care system.

Early recognition of cardiac arrest, prompt activation of emergency services, and immediate initiation of cardiopulmonary resuscitation (CPR) constitute the first two links in the chain of survival. Rapid defibrillation further increases the likelihood of return of spontaneous circulation (ROSC) in shockable rhythms. Fulfilment of the first—and increasingly the first three—links in the chain of survival depends on the knowledge and skills of bystanders as well as access to appropriate equipment. Post-resuscitation care, representing the final link, is typically provided by emergency medical services and continued in hospital settings [9]. Until the arrival of emergency medical teams, resuscitation efforts in primary care settings are performed by PHC staff.

Knowledge of emergency conditions and CPR skills is acquired during professional education for medical staff and during mandatory occupational health and safety training for non-medical staff. However, maintaining competence in high-quality CPR requires regular refresher training. Previous studies have shown that CPR quality among medical personnel declines as early as 3–6 months after training [10]. In Poland, there are no legal regulations mandating periodic CPR training for PHC staff, and training provision depends on decisions made by practice managers, who must bear organizational and financial costs. To date, no Polish study has comprehensively evaluated both guideline-based BLS knowledge and objectively measured CPR performance among PHC staff, including non-medical personnel with direct patient contact. In this context, there is a risk that both theoretical knowledge and practical skills may be insufficient, particularly with respect to chest compressions, which are regarded as the cornerstone of effective CPR. Simulation-based assessment allows standardized, objective evaluation of CPR quality without ethical and logistical constraints inherent to real-life OHCA events.

From a public health perspective, cardiac arrest in primary care represents a low-frequency but high-consequence event in which the quality of the initial response may depend on local workforce preparedness, access to equipment, and the presence of clear emergency procedures. In primary care settings, both medical and non-medical personnel may be involved in the first minutes of response, including recognition of cardiac arrest, activation of emergency medical services, initiation of chest compressions, and use of an automated external defibrillator. Therefore, assessing BLS preparedness across the whole PHC team is relevant not only to individual competence but also to patient safety and emergency preparedness at the health-system level.

This study aimed to evaluate basic life support preparedness among medical and non-medical primary health care staff by assessing guideline-based BLS knowledge and objectively measured chest compression performance. We also examined demographic, professional, and workplace-related factors associated with knowledge and CPR quality. By linking theoretical knowledge with simulation-based performance metrics, the study sought to identify modifiable gaps in the readiness of primary care teams to respond to cardiac arrest before the arrival of emergency medical services.

## 2. Materials and Methods

### 2.1. Study Design, Setting and Time Frame

A multicentre cross-sectional study was conducted between December 2024 and May 2025. The study aimed to assess knowledge and practical skills related to basic life support among staff working in selected primary health care practices located in the city of Poznań and Poznań County, Poland.

The study comprised two parallel components:

- A theoretical knowledge assessment using a multiple-choice questionnaire with a single correct answer (maximum score: 15 points, Supplementary File S2).
- An objective assessment of practical chest compression skills during a two-minute continuous compression trial.

The study was reported in accordance with the STROBE guidelines for cross-sectional studies (Supplementary File S1).

### 2.2. Selection of Primary Health Care Practices

The study was conducted in 16 primary health care practices located in the city of Poznań and Poznań County, Poland. A list of eligible practices was obtained from the official Ministry of Health registry. The study was designed as an exploratory multicentre assessment intended to include practices representing different population settings and organizational contexts rather than to achieve formal population representativeness. Therefore, no formal a priori sample size calculation was performed. The sample size was therefore determined pragmatically based on the number of participating practices, eligible staff availability, and feasibility of standardized simulation-based assessment.

Practices were stratified according to location into four categories: rural areas, towns with fewer than 20,000 inhabitants, towns with 20,000–100,000 inhabitants, and cities with more than 100,000 inhabitants. Within each stratum, practices were randomly selected using an online randomization tool (www.randomizer.org). Following selection, practice managers were contacted and invited to participate in the study. In cases of refusal, random selection was repeated within the same stratum until the planned number of participating practices was reached.

### 2.3. Participants

All staff members with direct patient contact working in the selected practices were invited to participate. Direct patient contact was defined as regular face-to-face interaction with patients in the practice, including medical consultation, nursing care, registration, or front-office duties. A total of 248 eligible staff members were invited, of whom 162 agreed to participate and completed the knowledge assessment. Participation within each practice depended on individual informed consent and availability. Therefore, the professional composition of participants could differ between practices and may not fully reflect the workforce structure of each participating practice. Consequently, the study was not designed to compare individual practices or to estimate practice-specific performance; practices were used as recruitment sites and, in the statistical analysis, as clustering units reflecting the multicentre structure of the data. The study population included medical personnel (physicians, nurses, and midwives) as well as administrative staff. All participants provided informed consent and were informed of their right to withdraw from the study at any stage without consequences.

Participants who were unable to perform chest compressions due to medical contraindications (musculoskeletal disorders, cardiovascular disease, recent surgical procedures, or recent trauma) were excluded from the practical skills assessment component (n = 10).

### 2.4. Study Procedure

To minimize potential distractions related to routine clinical duties, all study sessions were conducted on Saturdays, when primary care practices do not provide standard medical services. The practical assessment of chest compression skills was performed in a separate room to ensure standardized conditions.

To reduce potential learning effects, the order of the two study components (knowledge test vs. practical skills assessment) was randomized for each participant using www.randomizer.org. Participants did not receive feedback regarding their performance in either component upon completion of the tasks.

All participants received identical standardized verbal instructions delivered by the same investigator to minimize variability in task interpretation.

### 2.5. Knowledge Assessment

An author-developed questionnaire was created based on current ERC guidelines and adapted to the context of primary health care practice.

The questionnaire consisted of two parts:

Part one (demographic section): demographic and professional data, previous training, prior experience with cardiac arrest events, and workplace characteristics.

Part two (knowledge test): questions addressing patient assessment, recognition of cardiac arrest, basic life support procedures, and use of an automated external defibrillator (AED).

Prior to the main study, the questionnaire was reviewed by two family physicians and two paramedics to assess content relevance and clinical accuracy. Subsequently, it was pilot-tested among staff from two primary health care practices (n = 14) to evaluate clarity and comprehensibility. Minor linguistic revisions were implemented based on feedback from the pilot phase. The pilot phase was intended to assess clarity, comprehensibility, and feasibility of completion rather than to provide full psychometric validation. Practices participating in the pilot study were excluded from the final analysis. The internal consistency of the knowledge questionnaire was assessed using Cronbach’s alpha, yielding a value of 0.67, indicating acceptable reliability for an exploratory knowledge assessment but also indicating that the questionnaire should not be regarded as fully validated.

The questionnaire is provided as Supplementary File S2. The English version included as Supplementary Material is a linguistically edited translation of the questionnaire used in the study; the substantive meaning of the items and response options was preserved.

### 2.6. Practical Skills Assessment

Chest compression quality was assessed using a Resusci Anne QCPR manikin (Laerdal, Norway). Data were recorded using a Sim Pad Skill Reporter system (Laerdal) with up-to-date software designed for objective measurement of CPR quality parameters.

After receiving standardized instructions, the investigator left the room, and the participant initiated uninterrupted chest compressions at a self-selected time point. After two minutes, the investigator re-entered the room and informed the participant that the assessment was complete.

Prior to each study day, the equipment was tested to ensure proper functioning.

The following parameters were analysed:

- hand position during compressions;
- mean compression depth;
- percentage of compressions within the recommended depth range (50–60 mm);
- percentage of compressions with full chest recoil;
- mean compression rate;
- percentage of compressions within the recommended rate range (100–120/min);
- QCPR score.

The QCPR score was generated automatically by the Skill Reporter software version 7.3.8042 as a composite indicator of chest compression quality ranging from 0 to 100, with higher values indicating better overall chest compression performance. In this compression-only assessment, the score reflected guideline-based parameters measured by the manikin and software, including compression depth, compression rate, chest release, and hand position. Chest compression fraction was not analysed separately because all participants performed uninterrupted compressions for two minutes. Because the exact weighting of individual score components is not provided in the software output, separate chest compression quality parameters were also reported.

Chest compression quality parameters were defined according to European Resuscitation Council guidelines. Correct compression depth was defined as 50–60 mm, and correct compression rate as 100–120 compressions per minute.

### 2.7. Bias

Potential sources of bias included selection bias related to voluntary participation, performance bias due to the simulated setting, and the Hawthorne effect. Additionally, participants’ prior training and physical fitness were not controlled for and may have influenced performance.

### 2.8. Statistical Analysis

The normality of data distribution for all continuous variables was assessed using the Shapiro–Wilk test. Variables with a normal distribution were summarized using the mean and standard deviation (SD), whereas variables that did not follow a normal distribution were presented as the median with interquartile range [IQR]. Categorical variables were reported as percentages and absolute numbers (n).

Given the distribution of the analysed variables, correlations between CPR quality parameters and continuous variables (age and years of professional experience) were assessed using Spearman’s rank correlation coefficient.

Differences in CPR quality parameters between professional groups were analysed using the Kruskal–Wallis test followed by Dunn–Bonferroni post hoc comparisons.

All statistical tests were two-tailed, and a *p*-value of <0.05 was considered statistically significant. Statistical analyses were performed using IBM SPSS Statistics version 29.0, (IBM Corp., Armonk, NY, USA). To identify independent predictors of BLS knowledge and CPR quality, multivariable linear regression analyses were performed. Two separate models were constructed: one with the BLS knowledge score as the dependent variable (n = 162) and one with QCPR score as the dependent variable (n = 152). Participants with missing QCPR data were excluded only from the CPR quality model. No imputation of missing data was performed.

Covariates included age, time since graduation, profession, prior occupational exposure to cardiac arrest, and place of practice. Because only one midwife participated in the study, nurses and the midwife were combined into a single category for group comparisons and regression analyses. Multicollinearity was assessed using variance inflation factors (VIFs) and tolerance values for predictors included in the multivariable regression models. VIF values above 5 and tolerance values below 0.20 were considered to indicate potentially relevant multicollinearity. Due to a strong correlation between age and time since graduation, these variables were retained in the model, but their interpretation was performed with caution. Because participants were recruited from 16 primary health care practices, sensitivity analyses were performed to account for clustering at the practice level. Mixed-effects linear regression models with a random intercept for practice were fitted for the two main outcomes: BLS knowledge score and QCPR score. These models included the same fixed effects as the primary multivariable models. Intraclass correlation coefficients were calculated to quantify residual between-practice variability. Practice was treated as a clustering variable rather than a unit of comparison, because only staff members who agreed to participate were assessed in each practice.

Regression results were reported as β coefficients with 95% confidence intervals.

### 2.9. Use of Generative Artificial Intelligence

Generative artificial intelligence tools were used only for language editing. The authors reviewed and edited the final text and take full responsibility for the manuscript.

## 3. Results

### 3.1. Participant Characteristics

**Figure 1:**
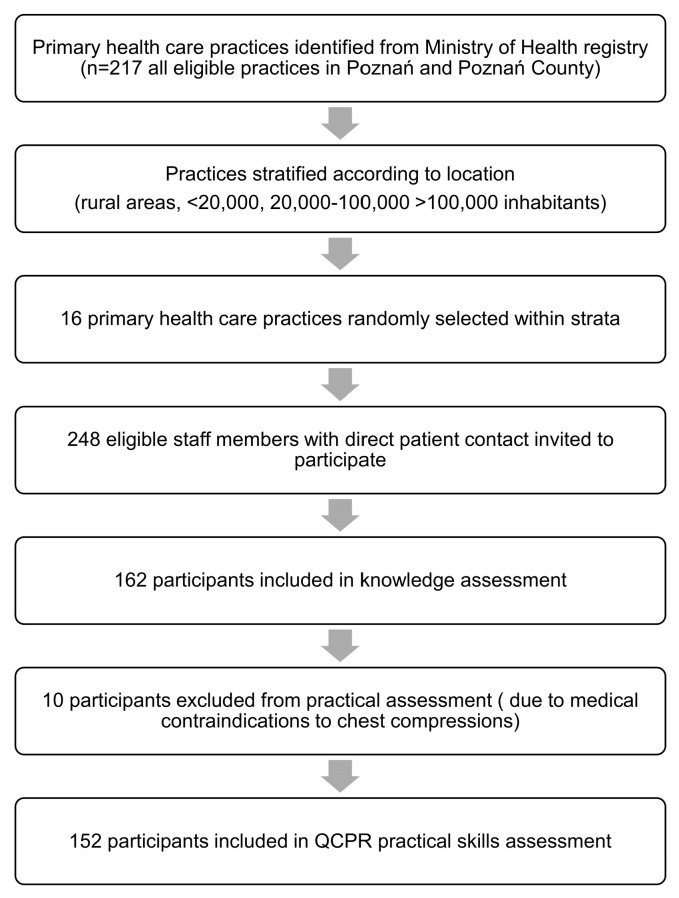
Flow diagram of participant recruitment and inclusion in the study. QCPR—quality cardiopulmonary resuscitation.

A total of 248 eligible staff members with direct patient contact working in the selected primary health care practices were invited to participate in the study. Of these, 162 agreed to participate and completed the knowledge assessment (response rate: 65.3%). Reasons for non-participation among the 86 eligible staff members who did not take part in the study were not systematically collected. Ten participants were excluded from the practical assessment because of medical contraindications to chest compressions, resulting in 152 participants included in the practical QCPR assessment. The number and professional composition of participants varied between practices. Because participation was incomplete within practices, practice-level comparisons were not performed. Participant recruitment and inclusion in the study are presented in Figure 1.

### 3.2. BLS Knowledge

The study group comprised 60 physicians, 60 nurses, one midwife, and 41 administrative staff members. Women accounted for 98.1% of the study population (n = 159). The mean age was 50.7 years, and the mean time since completion of formal education was 25.6 years. Participation in postgraduate training was reported by 42.6% of respondents, while 41.4% reported previous occupational exposure to a cardiac arrest event. Previous BLS training was reported by 41 participants (25.3%), whereas 121 participants (74.7%) reported no previous BLS/CPR training. Regarding training recency, no participant had completed BLS/CPR training within the preceding year, two participants (1.2%) had completed training 1–2 years before the study, and 39 participants (24.1%) had completed training more than 2 years before the study. Detailed characteristics of the study group are presented in Table 1.

The median score on the BLS knowledge test was 9/15 points.

Overall, the level of theoretical knowledge related to basic life support varied considerably across individual questionnaire items. The highest proportion of correct responses was observed for questions addressing the correct cardiopulmonary resuscitation sequence in adults, which was correctly identified by 91.4% of participants. Similarly, a high level of correctness was noted for identifying the highest priority during cardiac arrest management (84.0%) and recognizing contraindications to automated external defibrillator (AED) use in the primary care setting (75.9%).

Moderately high rates of correct responses were observed for the initial action after assessing unresponsiveness (71.0%), appropriate timing of AED use during resuscitation (67.9%), and correct first action after cessation of convulsions (61.7%). More than half of the participants correctly identified the appropriate management of agonal breathing (56.2%) and the recommended chest compression depth in adults (56.8%).

In contrast, several critical elements of basic life support demonstrated low levels of correct responses. Correct hand placement during chest compressions was identified by only 30.3% of participants, representing the lowest level of knowledge among all assessed items. Similarly, fewer than one-third of respondents correctly identified the appropriate action after confirming unresponsiveness in the presence of bystanders (29.0%). Knowledge regarding the recommended frequency of rescuer changes during cardiopulmonary resuscitation was also limited, with only 43.8% of participants providing a correct response.

Notably, only approximately half of the participants correctly identified the recommended chest compression rate (50.0%), duration of breathing and pulse assessment (50.6%), and correct placement of AED electrodes (50.6%), indicating substantial gaps in knowledge related to core resuscitation skills. Complete results were summarized in Table 2.

### 3.3. Simulated Chest Compression Performance

The mean chest compression depth was 41.3 mm, remaining below the recommended range of 50–60 mm. Only 23.5% of compressions met guideline-recommended depth criteria. Correct compression rate (100–120/min) was maintained in 30.2% of compressions. Full chest recoil was observed in 55.0% of attempts, while correct hand positioning was achieved in 82.6% of compressions. Reported percentages represent participant-level averages.

A Spearman rank correlation analysis demonstrated a statistically significant weak negative association between participant age and mean chest compression rate (Spearman’s ρ = −0.254, *p* = 0.0016), indicating that older participants tended to perform chest compressions at lower rates. A similar weak negative association was observed between years since graduation and mean compression rate (Spearman’s ρ = −0.234, *p* = 0.0036). Additionally, a moderate negative association was found between participant age and knowledge test score (Spearman’s ρ = −0.475, *p* < 0.001).

#### Professional Group-Specific Analysis

Overall differences between professional groups were statistically significant for QCPR score (*p* < 0.001), mean compression depth (*p* = 0.040), correct hand position (*p* = 0.027), and percentage of compressions with correct depth (*p* = 0.026). In Dunn–Bonferroni post hoc comparisons, QCPR score differed significantly between physicians and nurses (*p* = 0.0139) and between physicians and administrative staff (*p* < 0.001). The percentage of compressions with correct depth differed significantly between physicians and administrative staff (*p* = 0.0249). No significant post hoc differences were identified for mean compression depth, correct hand position, full chest recoil, correct compression rate, or mean compression rate. Detailed chest compression performance parameters stratified by profession are presented in Table 3.

### 3.4. Multivariable Analysis

In multivariable linear regression analysis, profession and practice location were independent predictors of BLS knowledge score (Table 4). Compared with physicians, nurses achieved lower scores (β = −1.0, *p* = 0.025), while administrative staff demonstrated substantially lower performance (β = −3.3, *p* < 0.001). Participants working in urban settings (20,000–100,000 and >100,000 inhabitants) achieved significantly higher knowledge scores compared with those working in rural areas.

Age was negatively associated with knowledge score (β = −0.1, *p* = 0.047), while time since graduation showed a borderline association (β = −0.1, *p* = 0.057). Due to a strong correlation between these variables, their independent effects should be interpreted with caution.

Multicollinearity diagnostics showed moderate collinearity between age and time since graduation. VIF values for age and time since graduation were 5.59 and 5.05 in the BLS knowledge model and 5.59 and 5.08 in the QCPR score model, respectively. Corresponding tolerance values were close to or slightly below 0.20; therefore, the independent effects of these variables should be interpreted with caution.

In the model assessing predictors of CPR quality (Table 5), higher BLS knowledge score was independently associated with better QCPR performance (β = 2.2 per point increase, *p* = 0.036). Administrative staff demonstrated significantly lower QCPR scores compared with physicians (β = −20.7, *p* = 0.007). No significant associations were observed for age, time since graduation, place of practice, or prior exposure to cardiac arrest.

In sensitivity analyses accounting for clustering by primary health care practice, mixed-effects linear regression models with a random intercept for practice were fitted for both main outcomes. The conditional intraclass correlation coefficient was 0.096 for BLS knowledge score and 0.068 for QCPR score, indicating modest residual clustering by practice. The main associations for BLS knowledge score remained broadly consistent with the participant-level analysis. For QCPR score, administrative staff continued to have significantly lower scores than physicians, whereas the association between BLS knowledge score and QCPR score was attenuated and no longer statistically significant after accounting for practice-level clustering. The results of the mixed-effects sensitivity analyses are presented in Table 6.

## 4. Discussion

This multicentre cross-sectional study identified important gaps in BLS knowledge and chest compression performance among medical and non-medical primary health care staff. In this study, the term “multicentre” refers to recruitment from multiple independent PHC practices rather than to coverage of multiple administrative regions or nationally representative centres. The results suggest that emergency preparedness in primary care may be insufficient during the critical interval before emergency medical services arrive. The principal contribution of this study is the combined assessment of guideline-based BLS knowledge and objective QCPR-measured chest compression quality in a PHC workforce sample.

The additional mixed-effects sensitivity analyses showed that clustering by practice had some impact on the interpretation of individual-level associations. Although the main descriptive findings remained unchanged, the association between BLS knowledge score and QCPR performance observed in participant-level regression was attenuated after accounting for practice-level clustering. This suggests that simulated CPR performance in PHC staff may depend not only on individual theoretical knowledge but also on shared practice-level factors, such as local training culture, emergency procedures, availability of equipment, staffing patterns, and organizational priorities.

The most striking finding of the practical skills assessment was the inadequate quality of chest compressions, particularly with respect to compression depth and rate. The mean compression depth observed in this study remained well below guideline-recommended values, with fewer than one-quarter of compressions meeting depth criteria. This is of major concern, as insufficient compression depth has been consistently associated with reduced coronary and cerebral perfusion pressures and poorer survival outcomes following cardiac arrest [11]. Similarly, correct compression rate was maintained in less than one-third of compressions, indicating difficulties in simultaneously achieving both key parameters of high-quality CPR. The observed negative association between participant age and both chest compression rate and overall knowledge score warrants particular attention. While age itself is not a modifiable factor, this finding likely reflects longer intervals since initial training and less frequent exposure to structured resuscitation education among more experienced staff.

Previous Polish data on CPR knowledge among PHC staff are scarce. Tomaszek et al. reported insufficient CPR knowledge in 34% of nurses, including those working in PHC settings [12]. Similar deficiencies in BLS knowledge among PHC personnel have been reported in other European countries [13,14]. Poor chest compression quality during simulated CPR performed by PHC staff has also been described in Spain [15], as well as in studies conducted in Brazil [16] and Nepal [17]. These findings suggest that insufficient CPR competence among PHC staff is a global issue rather than a country-specific problem.

Concerns regarding inadequate CPR skills in general practice were already raised in the 1990s, when Hollis and Gillespie emphasized the need for structured training due to poor resuscitation performance among PHC personnel [18].

The discrepancy between selected knowledge items and objectively measured chest compression performance indicates that declarative knowledge of BLS algorithms may be insufficient to ensure effective practical response. For PHC systems, this distinction is important because emergency preparedness requires not only awareness of guidelines but also the ability to perform high-quality chest compressions under time pressure, physical exertion, and local organizational constraints. These findings support the need for recurrent, practice-based BLS training that incorporates objective feedback and includes all staff members likely to participate in the initial response to cardiac arrest.

In Poland, the median response time of emergency medical services is measured from the receipt of the call by the emergency medical dispatcher to the arrival of the emergency medical team at the scene and should not exceed 8 min in cities with more than 10,000 inhabitants and 15 min in areas with fewer than 10,000 inhabitants [19]. According to 2025 data from the study area, these response-time thresholds were exceeded, reaching approximately 12 and 17 min, respectively [20]. During this interval, resuscitation is performed by witnesses to the event, including PHC personnel. Survival after OHCA remains low; however, when cardiac arrest occurs in a primary care practice, outcomes may improve, provided that staff are adequately trained and an automated external defibrillator (AED) is available and used before the arrival of emergency medical services [8,21]. From a systems perspective, the findings highlight a missed opportunity to improve OHCA outcomes. Cardiac arrest occurring in primary care settings is associated with higher survival rates compared with arrests in other community locations, provided that early CPR and defibrillation are promptly initiated. Ensuring that PHC teams possess and maintain adequate resuscitation competence may therefore represent a highly effective, yet underutilized, public health intervention.

The moderate level of knowledge and low CPR quality observed in this study may partly reflect the lack of mandatory, recurrent BLS training in PHC settings. While hospital staff often undergo annual CPR training due to accreditation requirements, no analogous obligations exist for PHC practices. Current Polish regulations require employers to designate individuals trained in first aid but do not mandate comprehensive or recurrent BLS training for all staff [22]. Consequently, the resuscitation potential of PHC teams remains underutilized.

Taken together, the results support the need for structured, recurrent, and simulation-based BLS training programs tailored to the primary care environment. Training initiatives should encompass not only medical staff but also administrative personnel, who may be first responders in the event of a cardiac arrest within a primary care facility. The integration of objective feedback technologies, such as QCPR systems, may further enhance skill acquisition and retention by providing immediate, performance-based feedback.

### Limitations

Several limitations of this study should be acknowledged. First, the study was conducted in a single metropolitan region and its surrounding county, which may limit the generalizability of the findings to other geographic areas, particularly regions with different organizational structures or access to training resources. Therefore, the multicentre nature of the study should be understood as involving multiple practice sites within one metropolitan region and surrounding county, not as evidence of national geographic representativeness. Additionally, voluntary participation may have introduced non-response bias. Staff members who agreed to participate may have differed from non-participants in motivation, confidence, previous training, or perceived competence. The absence of a formal a priori sample size calculation limits the interpretation of non-significant findings, particularly for small effects and subgroup comparisons.

Second, the study population was predominantly female, with women accounting for 98.1% of participants. This distribution likely reflects the sex structure of the participating PHC workforce, particularly the high proportion of nurses and administrative staff. However, it limits the ability to generalize the findings to male PHC staff and precludes meaningful sex-specific comparisons. Because physical characteristics and fatigue may influence chest compression performance, future studies should include more sex-balanced samples or collect additional anthropometric variables to better assess their relationship with CPR quality.

Third, the cross-sectional design precludes conclusions regarding causality or longitudinal skill retention. The study provides a snapshot of current knowledge and performance but does not assess how skills evolve over time or in response to training interventions.

Fourth, the knowledge assessment was based on an author-developed questionnaire. Although the tool underwent expert review, pilot testing, and internal consistency assessment, it was not subjected to full psychometric validation, such as test–retest reliability, factor analysis, or validation against an established BLS knowledge instrument. Therefore, the results should be interpreted as an exploratory assessment of guideline-based theoretical knowledge rather than a definitive measure of clinical competence.

Fifth, the practical assessment focused exclusively on chest compression quality. Other critical components of basic life support, including ventilation technique, effective use of an automated external defibrillator, teamwork, communication, and role allocation, were not evaluated. Consequently, the overall quality of resuscitation performance may have been overestimated or underestimated.

Sixth, CPR performance was assessed in a simulated environment, which may not fully reflect real-life behaviour during an actual cardiac arrest. Factors such as emotional stress, environmental constraints, and team dynamics, which can significantly influence performance, were not replicated. Additionally, awareness of being observed may have introduced a Hawthorne effect, potentially leading to better performance than would occur in routine clinical practice.

Finally, differences in motivation, physical fitness, and prior exposure to cardiac arrest events among professional groups were not systematically controlled for and may have influenced the observed results.

Despite these limitations, the study provides valuable, objective insight into the preparedness of PHC staff to manage cardiac arrest and underscores the need for further nationwide research and targeted educational interventions.

Additionally, a high degree of collinearity between age and time since graduation was observed, which may have affected the stability of regression estimates for these variables.

## 5. Conclusions

Primary health care staff demonstrated insufficient knowledge of BLS and low quality of simulated CPR, particularly with respect to chest compression depth and rate. These findings suggest that emergency preparedness in primary care may be insufficient and support the implementation of structured, recurrent, simulation-based BLS training for both medical and non-medical PHC personnel.

## Figures and Tables

**Table 1:** Demographic and professional characteristics of PHC staff participating in the study (n = 162).

Variable	n (%)
**Sex**	
Female	159 (98.1)
Male	3 (1.9)
**Profession**	
Physician	60 (37.0)
Nurse	60 (37.0)
Midwife	1 (0.6)
Administrative staff	41 (25.3)
**Education level**	
Secondary education	63 (38.9)
Bachelor’s degree	16 (9.9)
Master’s degree	23 (14.2)
Medical degree (MD)	57 (35.2)
PhD (medical sciences)	3 (1.9)
**Professional specialization status**	
Specialist training in progress	2 (1.2)
Completed specialization	67 (41.4)
No postgraduate training	93 (57.4)
**Number of previous CPR attempts in clinical practice**	
0	95 (58.6)
1	27 (16.7)
2	26 (16.0)
3	12 (7.4)
≥4	2 (1.2)
**Previous BLS training**	
**Yes**	41 (25.3)
No	121 (74.7)
**Time since last BLS training**	
**<1 year**	0 (0.0)
**1–2 years**	2 (1.2)
**>2 years**	39 (24.1)
**Never**	121 (74.7)
**Place of work (population size)**	
Rural areas	47 (29.0)
<20,000 inhabitants	28 (17.3)
20,000–100,000 inhabitants	38 (23.5)
>100,000 inhabitants	49 (30.3)

Note: Data are presented as n (%). PHC, primary health care; CPR, cardiopulmonary resuscitation; BLS, basic life support; MD, medical degree; PhD, doctoral degree in medical sciences. Percentages may not total 100% because of rounding.

**Table 2:** Performance on individual items of the BLS knowledge assessment questionnaire (n = 162).

Questionnaire Item	Correct, n (%)
First action after assessing unresponsiveness	115 (71.0)
Correct action after confirming unresponsiveness in the presence of bystanders	47 (29.0)
Correct management of agonal breathing	91 (56.2)
Correct duration of breathing/pulse assessment	82 (50.6)
Correct chest compression rate	81 (50.0)
Correct chest compression depth in adults	92 (56.8)
Correct hand placement during chest compressions	49 (30.3)
Correct CPR sequence in adults	148 (91.4)
Highest priority during cardiac arrest management	136 (84.0)
Correct timing of AED use during CPR	110 (67.9)
Correct identification of contraindications to AED use in primary care	123 (75.9)
Correct indication to interrupt chest compressions	89 (54.9)
Correct placement of AED electrodes	82 (50.6)
Correct frequency of rescuer changes during CPR (if resources allow)	71 (43.8)
Correct first action after cessation of convulsions	100 (61.7)

Note: Data are presented as n (%). Each item had one correct answer. BLS, basic life support; CPR, cardiopulmonary resuscitation; AED, automated external defibrillator.

**Table 3:** Chest compression performance by profession.

	Profession			
Variable	Physicians	Nurse/Midwife	Administrative Staff	Dunn–Bonferroni Post Hoc *p*-Values
QCPR Score	36.5 [13.5–65.5]	12.0 [0.0–41.5]	9.0 [0.0–21.0]	† 0.0139
				§ < 0.001
				* 0.3714
Mean depth [mm]	43.7 (9.6)	40.3 (10.2)	39.1 (9.8)	† 0.1938
				§ 0.0526
				* 1
Correct hand position [%]	100.0 [100.0–100.0]	100.0 [62.5–100.0]	100.0 [82.5–100.0]	† 0.0910
				§ 0.0520
				* 1
Full recoil [%]	78.0 [21.0–98.0]	42.5 [13.25–91.0]	61.0 [13.5–95.5]	† 0.8316
				§ 1
				* 1
Correct depth [%]	19.0 [0.0–60.5]	1.0 [0.0–41.75]	0.0 [0.0–10.5]	† 0.2652
				§ 0.0249
				* 0.8743
Correct rate [%]	35.5 [1.0–76.0]	2.0 [0.0–55.3]	2.5 [0.0–39.5]	† 0.3059
				§ 0.1239
				* 1
Mean rate [min^−1^]	106.4 (20.4)	102.6 (27.9)	107.1 (30.9)	† 1
				§ 1
				* 1

Values are presented as median [IQR] or mean (SD), as appropriate. *p*-value for Dunn–Bonferroni post hoc test: † physician vs. nurses, § physician vs. administration, * nurses vs. administration.

**Table 4:** Multivariable linear regression for BLS knowledge score (n = 162).

Variable	β	95% CI	*p*-Value
Nurse/midwife vs. physician	**−1.0**	**−1.9 to −0.1**	**0.025**
Administrative staff vs. physician	**−3.3**	**−4.3 to −2.3**	**<0.001**
Town/city < 20,000 vs. rural	0.9	−0.2 to 1.9	0.115
Town/city 20,000–100,000 vs. rural	**1.6**	**0.6 to 2.6**	**0.002**
City > 100,000 vs. rural	**1.3**	**0.4 to 2.3**	**0.004**
Age, per year	**−0.1**	**−0.1 to 0.0**	**0.047**
Time since graduation, per year	−0.1	−0.1 to 0.0	0.057
Prior CPR at work, yes vs. no	−0.7	−1.5 to 0.1	0.098

Note: β coefficients represent adjusted differences in BLS knowledge score. The BLS knowledge score ranged from 0 to 15 points, with higher scores indicating better knowledge. Reference categories were physician for profession and rural area for place of practice. CI, confidence interval; BLS, basic life support; CPR, cardiopulmonary resuscitation. Bold values indicate statistically significant associations (*p* < 0.05).

**Table 5:** Multivariable linear regression for QCPR score (n = 152).

Variable	β	95% CI	*p*-Value
Nurse/midwife vs. physician	−8.8	−20.3 to 2.7	0.133
Administrative staff vs. physician	**−20.7**	**−35.6 to −5.8**	**0.007**
Town/city < 20,000 vs. rural	−0.6	−14.6 to 13.4	0.931
Town/city 20,000–100,000 vs. rural	5.8	−7.4 to 19.1	0.386
City > 100,000 vs. rural	10.0	−2.2 to 22.3	0.108
Age, per year	0.1	−0.8 to 1.0	0.856
Time since graduation, per year	0.1	−0.7 to 0.9	0.835
BLS knowledge score, per point	**2.2**	**0.1 to 4.3**	**0.036**
Prior CPR at work, yes vs. no	−6.1	−17.1 to 4.9	0.274

Note: β coefficients represent adjusted differences in QCPR score. The QCPR score ranged from 0 to 100, with higher values indicating better simulated chest compression performance. Reference categories were physician for profession and rural area for place of practice. CI, confidence interval; QCPR, quality cardiopulmonary resuscitation; BLS, basic life support; CPR, cardiopulmonary resuscitation. Bold values indicate statistically significant associations (*p* < 0.05).

**Table 6:** Mixed-effects sensitivity analyses accounting for clustering by primary health care practice.

Variable	BLS Knowledge Score β	95% CI	*p*-Value	QCPR Score β	95% CI	*p*-Value
Nurse/midwife vs. physician	−1.17	−2.00 to −0.33	**0.006**	−9.99	−21.38 to 1.39	0.085
Administrative staff vs. physician	−3.25	−4.24 to −2.26	**<0.001**	−22.38	−37.17 to −7.59	**0.003**
Town/city < 20,000 vs. rural	0.95	−0.41 to 2.32	0.171	−0.03	−16.77 to 16.71	0.997
Town/city 20,000–100,000 vs. rural	1.50	0.20 to 2.81	**0.024**	6.46	−9.74 to 22.66	0.434
City > 100,000 vs. rural	1.48	0.15 to 2.82	**0.030**	11.42	−4.86 to 27.70	0.169
Age, per year	−0.05	−0.12 to 0.01	0.107	−0.00	−0.87 to 0.87	0.998
Time since graduation, per year	−0.06	−0.12 to −0.01	**0.025**	0.12	−0.64 to 0.89	0.751
Prior CPR at work, yes vs. no	−0.52	−1.33 to 0.29	0.205	−5.30	−16.19 to 5.59	0.340
BLS knowledge score, per point	—	—	—	1.74	−0.45 to 3.93	0.118

Note: Mixed-effects linear regression models included a random intercept for primary health care practice. Fixed effects were identical to those included in the primary participant-level regression models. Practice was included as a clustering variable and was not interpreted as a unit of comparison. Reference categories were physician for profession and rural area for place of practice. CI, confidence interval; BLS, basic life support; CPR, cardiopulmonary resuscitation; QCPR, quality cardiopulmonary resuscitation. Bold values indicate statistically significant associations (*p* < 0.05).

## Data Availability

The data presented in this study are available upon request from the corresponding author.

## Supplementary Materials

The following supporting information can be downloaded at https://www.mdpi.com/article/10.3390/jcm15124460/s1, Supplementary File S1: STROBE checklist for cross-sectional studies (.docx); Supplementary File S2: Basic life support knowledge questionnaire (.docx).

## Abbreviations

The following abbreviations are used in this manuscript:

OHCA	out-of-hospital cardiac arrest
PHC	primary health care
BLS	basic life support
ERC	European Resuscitation Council
CPR	cardiopulmonary resuscitation
AED	automated external defibrillator
SD	standard deviation

## References

[1] Mani G., Annadurai K., Danasekaran R. (2015). Bystander cardiopulmonary resuscitation in out-of-hospital cardiac arrest: Need of the hour. Afr. Health Sci..

[2] Empana J.P., Lerner I., Valentin E., Folke F., Böttiger B., Gislason G., Jonsson M., Ringh M., Beganton F., Bougouin W. (2022). Incidence of sudden cardiac death in the European Union. J. Am. Coll. Cardiol..

[3] Gach D., Nowak J.U., Krzych Ł.J. (2016). Epidemiology of out-of-hospital cardiac arrest in the Bielsko-Biala district: A 12-month analysis. Pol. Heart J. (Kardiol. Pol.).

[4] Hjärtstam N., Rawshani A., Hellsén G., Råmunddal T. (2023). Comorbidities prior to out-of-hospital cardiac arrest and diagnoses at discharge among survivors. Open Heart.

[5] Statistics Poland (2024). Ambulatory Health Care in 2024.

[6] Eurostat (2022). Persons Visiting a Doctor in the Last 12 Months by Medical Speciality, Number of Visits, Educational Attainment Level, Sex and Age.

[7] Zylyftari N., Møller S.G., Wissenberg M., Folke F., Barcella C.A., Møller A.L., Gnesin F., Mills E.H.A., Jensen B., Lee C.J. (2021). Contacts with the health care system before out-of-hospital cardiac arrest. J. Am. Heart Assoc..

[8] Colquhoun M.C. (2014). Resuscitation in general practice—Time for action. Resuscitation.

[9] Merchant R.M., Becker L.B., Brooks S.C., Chan P.S., Del Rios M., McBride M.E., Neumar R.W., Previdi J.K., Uzendu A., Sasson C. (2024). The American Heart Association Emergency Cardiovascular Care 2030 impact goals and call to action to improve cardiac arrest outcomes. Circulation.

[10] Yang C.W., Yen Z.S., McGowan J.E., Chen H.C., Chiang W.C., Mancini M.E., Soar J., Lai M.S., Ma M.H.M. (2012). A systematic review of retention of adult advanced life support knowledge and skills in healthcare providers. Resuscitation.

[11] Kleinman M.E., Buick J.E., Huber N., Idris A.H., Levy M., Morgan S.G., Nassal M.M.J., Neth M.R., Norii T., Nunnally M.E. (2025). Part 7: Adult basic life support: 2025 American Heart Association Guidelines for Cardiopulmonary Resuscitation and Emergency Cardiovascular Care. Circulation.

[12] Tomaszek L., Cepuch G., Turkanik E. (2016). Assessment of the selected factors determining the level of knowledge of the team of nurses on cardiopulmonary resuscitation. Anestezjol. I Ratow..

[13] Secher N., Mikkelsen M.M., Adelborg K., Mikkelsen R., Grove E.L., Rubak J.M., Vedsted P., Løfgren B. (2012). Direct mail improves knowledge of basic life support guidelines in general practice: A randomised study. Scand. J. Trauma Resusc. Emerg. Med..

[14] Flores M.F.S., Alises I.C., Enríquez T.M., Gil T.C., González P.N. (2020). Nivel de conocimientos en reanimación cardiopulmonar de profesionales sanitarios de atención primaria de Valladolid Este y sus determinantes asociados. Med. Gen..

[15] Moreno S., Sisó-Almirall A., Kostov B., Expósito M., Moreno J.R., de Pablo B., Coll-Vinent B. (2021). Cardiopulmonary resuscitation skill maintenance for primary care staff: Brief training sessions with feedback. Emergencias.

[16] Meira Júnior L.E., Souza F.M., Almeida L.C., Veloso G.G.V., Caldeira A.P. (2016). Evaluation of basic life support training for physicians and nurses in primary care. Rev. Bras. Med. Fam. Comunidade.

[17] Chaudhary G.P., Sah K., Malla J., Das N., Chaudhary S., Chaudhary I., Pandey J. (2023). Knowledge regarding basic life support among health care workers of the hospital of Nepal. J. Healthc. Eng..

[18] Hollis S., Gillespie N. (2000). An audit of basic life support skills amongst general practitioner principals: Is there a need for regular training?. Resuscitation.

[19] Act of 8 September 2006 on the State Emergency Medical Services. https://isap.sejm.gov.pl/isap.nsf/DocDetails.xsp?id=WDU20061911410.

[20] Greater Poland Voivodeship Office in Poznań *Voivodeship Action Plan for the State Emergency Medical Services System Poznań*; Greater Poland Voivodeship Office in Poznań: Poznań, Poland, 2025. https://www.poznan.uw.gov.pl/wojewodzki-plan-dzialania-systemu-prm.

[21] Haskins B., Nehme Z., Cameron P.A., Smith K. (2021). Cardiac arrests in general practice clinics or witnessed by emergency medical services: A 20-year retrospective study. Med. J. Aust..

[22] Regulation of the Minister of Labour and Social Policy of 26 September 1997 on General Occupational Health and Safety Provisions. https://isap.sejm.gov.pl/isap.nsf/DocDetails.xsp?id=wdu19971290844.

