# Supplementary material for: Basic Life Support Knowledge and Simulated Chest Compression Performance Among Primary Health Care Staff: A Multicentre Cross-Sectional Study
Source: J Clin Med. 2026 Jun 9;15(12):4460. doi: 10.3390/jcm15124460 (PMC13302723; doi:10.3390/jcm15124460)
Supplement: Supplementary file 1 [file jcm-15-04460-s001.zip › Additional file 1 STROBE checklist for cross-sectional studies.pdf]

Additional file 1: STROBE checklist for cross-sectional studies

	Item No	Recommendation	Page No
**Title and abstract**	1	(a) Indicate the study's design with a commonly used term in the title or the abstract	1
		(b) Provide in the abstract an informative and balanced summary of what was done and what was found	1
**Introduction**			
**Background/rationale**	2	Explain the scientific background and rationale for the investigation being reported	2
**Objectives**	3	State specific objectives, including any prespecified hypotheses	3
**Methods**			
**Study design**	4	Present key elements of study design early in the paper	3
**Setting**	5	Describe the setting, locations, and relevant dates, including periods of recruitment, exposure, follow-up, and data collection	3
**Participants**	6	(a) Give the eligibility criteria, and the sources and methods of selection of participants	3–4
		(b) For matched studies, give matching criteria and number of exposed and unexposed	Not applicable
**Variables**	7	Clearly define all outcomes, exposures, predictors, potential confounders, and effect modifiers. Give diagnostic criteria, if applicable	3–5
**Data sources/measurement**	8*	For each variable of interest, give sources of data and details of methods of assessment/measurement. Describe comparability of assessment methods if there is more than one group	3–5, Additional file 2
**Bias**	9	Describe any efforts to address potential sources of bias	5, 12
**Study size**	10	Explain how the study size was arrived at	3

**Quantitative variables**	11	Explain how quantitative variables were handled in the analyses. If applicable, describe which groupings were chosen and why	4–5
**Statistical methods**	12	(a) Describe all statistical methods, including those used to control for confounding	5
		(b) Describe any methods used to examine subgroups and interactions	5
		(c) Explain how missing data were addressed	5
		(d) If applicable, describe analytical methods taking account of sampling strategy	3,5
		(e) Describe any sensitivity analyses	5
**Results**			
**Participants**	13*	(a) Report numbers of individuals at each stage of study — e.g. numbers potentially eligible, examined for eligibility, confirmed eligible, included in the study, completing follow-up, and analysed	5–6
		(b) Give reasons for non-participation at each stage	5–6
		(c) Consider use of a flow diagram	6
**Descriptive data**	14*	(a) Give characteristics of study participants and information on exposures and potential confounders	6–7
		(b) Indicate number of participants with missing data for each variable of interest	5
**Outcome data**	15*	Report numbers of outcome events or summary measures	8–10
**Main results**	16	(a) Give unadjusted estimates and, if applicable, confounder-adjusted estimates and their precision, e.g. 95% confidence interval. Make clear which confounders were adjusted for and why they were included	9–10
		(b) Report category boundaries when continuous variables were categorized	3–5, 7
		(c) If relevant, consider translating estimates of relative risk into absolute risk for a meaningful time period	Not applicable

**Other analyses**	17	Report other analyses done — e.g. analyses of subgroups and interactions, and sensitivity analyses	8–10
**Discussion**			
**Key results**	18	Summarise key results with reference to study objectives	10–11
**Limitations**	19	Discuss limitations of the study, taking into account sources of potential bias or imprecision. Discuss both direction and magnitude of any potential bias	12
**Interpretation**	20	Give a cautious overall interpretation of results considering objectives, limitations, multiplicity of analyses, results from similar studies, and other relevant evidence	10–12
**Generalisability**	21	Discuss the generalisability/external validity of the study results	12
**Other information**			
**Funding**	22	Give the source of funding and the role of the funders for the present study and, if applicable, for the original study on which the present article is based	13
