# Supplementary material for: Basic Life Support Knowledge and Simulated Chest Compression Performance Among Primary Health Care Staff: A Multicentre Cross-Sectional Study
Source: J Clin Med. 2026 Jun 9;15(12):4460. doi: 10.3390/jcm15124460 (PMC13302723; doi:10.3390/jcm15124460)
Supplement: Supplementary file 1 [file jcm-15-04460-s001.zip › Additional file 2 Basic life support knowledge questionnaire.pdf]

PARTICIPANT ID……..

PARTICIPANT CHARACTERISTICS

1. Sex:

- ☐ Female
- ☐ Male

2. Age:

………… years

3. Profession:

- ☐ Physician
- ☐ Nurse
- ☐ Midwife
- ☐ Administrative staff

4. Highest level of education:

- ☐ Secondary education
- ☐ Bachelor’s degree
- ☐ Master's degree
- ☐ Doctoral degree

5. Professional specialization status:

- ☐ No specialization
- ☐ Specialist training in progress
- ☐ Completed specialization
- ☐ Not applicable

6. Time since graduation/college:

………… years

7. Previous postgraduate BLS/ALS training:

- ☐ No
- ☐ Yes (Year of most recent training: ………., certified training? yes/no)

8. Previous occupational exposure to sudden cardiac arrest?

- ☐ No
- ☐ Yes (Number of previous events: …..)

9. Place of work:

- ☐ Rural area
- ☐ Town/city <20,000 inhabitants
- ☐ Town/city 20,000–100,000 inhabitants
- ☐ City >100,000 inhabitants

BLS KNOWLEDGE QUESTIONNAIRE

Instructions:

This questionnaire consists of multiple-choice questions. Each question has one correct answer. Please mark the letter corresponding to the correct answer. If you make a mistake, cross out the incorrect answer and clearly mark your final answer.

1. **A patient in the primary care practice suddenly loses consciousness and does not respond to voice or touch. What should be the first action?**
    a. Call emergency medical services
    b. Call loudly for help
    c. Start high-quality chest compressions
    d. Get an AED immediately
2. **After confirming that the patient is unresponsive and other people are present, what should be the next step?**
    a. Assess breathing and pulse
    b. Open the airway
    c. Start resuscitation immediately
    d. Place the patient in the recovery position
3. **If an unconscious patient is diagnosed with agonal breathing - single gasps resembling an attempt to take in air, the first thing to do is:**
    a. Place the patient in the recovery position
    b. Start cardiopulmonary resuscitation immediately
    c. Elevate the lower limbs and provide access to fresh air
    d. Prepare equipment and wait before starting cardiopulmonary resuscitation
4. **The breathing/pulse assessment of an unconscious patient should be performed during:**
    a. Up to 5 seconds
    b. Up to 10 seconds
    c. 10–20 seconds
    d. 30 seconds
5. **The recommended chest compression rate in adults is:**
    a. 60–80/min
    b. 80–100/min
    c. 100–120/min
    d. 120–140/min

6. The recommended chest compression depth in adults is:

a. 4–5 cm

b. 5–6 cm

c. 6–7 cm

d. 7–8 cm

7. When performing chest compressions in an adult, the hands should be placed:

a. In the centre of the chest

b. On the sternum at the nipple line

c. On the upper third of the sternum

d. Below the xiphoid process

8. Adult cardiopulmonary resuscitation should be performed:

a. In a sequence of 30 compressions: 2 rescue breaths

b. In a sequence of 15 compressions: 2 rescue breaths

c. Providing only chest compressions

d. In a sequence of 3 compressions: 1 rescue breath

9. The highest priority when managing a patient with sudden cardiac arrest is:

a. Using an Automated External Defibrillator

b. Obtaining intravascular access

c. Ventilation with a self-inflating bag

d. Providing high-quality chest compressions

10. When should an AED be used during cardiopulmonary resuscitation?

a. Immediately if available

b. After 2 minutes of cardiopulmonary resuscitation

c. After 3 minutes of cardiopulmonary resuscitation

d. After obtaining intravascular access

11. Which of the following is a contraindication to AED use in a primary care practice?

a. Pregnancy

b. Presence of an implanted pacemaker or cardioverter-defibrillator

c. Absence of a physician

d. None of the above

12. Chest compressions should be interrupted:

a. Every 3 minutes to assess vital signs
b. If there are signs of return of spontaneous circulation
c. To insert a supraglottic airway device
d. Immediately after turning on the AED to attach the electrode pads

13. The AED electrodes should be placed

a. One to the right of the sternum below the clavicle, the other in the left mid-axillary line at the level of the fifth intercostal space.
b. One under the left collarbone, the other near the apex of the heart.
c. Anywhere on the victim's chest
d. One under the right clavicle, the other in the left midclavicular line

14. Depending on available personnel, the person performing chest compressions should be changed:

a. No more than every 2 minutes
b. No more than every 4 minutes
c. Maximum every 2 minutes or more often if tired
d. Maximum every 5 minutes or more often if tired

15. The first action after cessation of a seizure episode in a patient who remains unconscious should be:

a. Opening the airway and assessing breathing
b. Placing the patient in the recovery position
c. Leaving the patient in the position in which they were found
d. Assessing for possible tongue injuries

*This questionnaire was developed for the purposes of the study to assess selected elements of guideline-based basic life support knowledge among primary health care staff. It is not a fully validated diagnostic instrument.

*This English version is a linguistically edited translation of the questionnaire used in the study. The wording was edited for clarity for publication, without changing the substantive meaning of the items or response options.
